# Prevalence and burden of anhedonia among patients with major depressive disorder in South Korea: A cross-sectional, observational study

**DOI:** 10.1371/journal.pone.0334525

**Published:** 2025-10-27

**Authors:** Bolam Lee, YoungDoe Kim, HeeKyung Eum, Ja Seo Koo, Keira Herr, Lawrence Vandervoort, Jung Goo Lee

**Affiliations:** 1 Johnson & Johnson, Seoul, South Korea; 2 Johnson & Johnson, Singapore; 3 Oracle Life Sciences, Singapore; 4 Department of Psychiatry, College of Medicine, Haeundae Paik Hospital, Inje University, Busan, Republic of Korea; 5 Paik Institute for Clinical Research, Inje University, Busan, Republic of Korea; Mayo Clinic College of Medicine and Science, UNITED STATES OF AMERICA

## Abstract

**Background:**

Anhedonia (ANH), a key symptom of major depressive disorder (MDD), has a substantial societal and economic burden. In South Korea, while MDD is prevalent, the evidence regarding anhedonia remains scarce. This study investigated the prevalence and impact of anhedonia in patients with MDD in South Korea, including patient and physician perceived goals and satisfaction with MDD treatment.

**Methods:**

This cross-sectional study (April-May 2023) included two surveys, one specific to patients (aged ≥18 years) with self-reported physician diagnosis of MDD and 9-item Patient Health Questionnaire (PHQ-9) score≥10, and another to physicians treating MDD. The MDD group was classified as MDD-ANH (Snaith-Hamilton Pleasure Scale [SHAPS] score≥3) and MDD non-ANH (SHAPS score≤2). Multiple regression models were employed to evaluate the effect of anhedonia on health-related quality of life (HRQoL), work productivity and activity impairment, and healthcare resource utilization (HCRU).

**Results:**

Of 4496 participants, the age- and gender-weighted prevalence of MDD was 9.9%, of which 61.5% self-reported anhedonia. Patients with MDD-ANH (vs. MDD non-ANH) had longer duration of depression since diagnosis and lower prior and current antidepressants use (all, p < 0.05). Similarly, patients with MDD-ANH (vs. MDD non-ANH) demonstrated lower HRQoL, and increased HCRU (all, p < 0.05). Patients with MDD-ANH prioritized treatment goal of improved sleep quality, while physicians prioritized avoiding suicidal thoughts. Similarly, patients with MDD-ANH had highest level of satisfaction with controlled depressed mood and physicians with improved sleep quality. Both groups reported lowest level of satisfaction with regaining interest in hobbies, regaining self-esteem, and improving sexual satisfaction.

**Conclusions:**

This is the first study in South Korea to estimate the prevalence of anhedonia in patients with MDD, highlighting the disease burden and unmet medical needs. Furthermore, disparities observed between patients and physicians in the goals and expectations of MDD treatment underscore the need to monitor anhedonia for treatment optimization.

## Introduction

Major depressive disorder (MDD) is a severe psychiatric condition that imposes a significant global burden, ranking as the leading contributor to disability-adjusted life years (29.6% in 2019) among all mental health disorders [1]. In South Korea, the prevalence of MDD has shown a consistent increase over time, as evidenced by epidemiological surveys and national insurance claims data, rising from 1.7% in 2001, 2.5% in 2006 to 5.3% in 2013 [2–5]. A national survey further revealed that MDD disproportionately affects specific demographic groups, with a progressive rise among women from low-income households and young men aged 18–29 [6].

Among the Organization for Economic Cooperation and Development (OECD) nations, South Korea reported the highest prevalence of depression (36.8%) in 2020 [7,8], with the figure at 17.7% in 2022 [9]. MDD is a significant risk factor for suicide, and South Korea has been reported to have the highest rates of suicide among the OECD countries [9,10].

Anhedonia, characterized as “lack of enjoyment from, engagement in, or energy for life’s experiences” and deficits in the capacity to experience pleasure or take interest in activities, is a central symptom of MDD and other psychological and neurological disorders [11–13]. Notably, anhedonia is reported in over 82% of patients with MDD [11,14] and is associated with severe cognitive and psychosocial impairment [15,16], greater depression severity [15,17], heightened suicidal ideation [18], reduced health-related quality of life (HRQoL) [19], and poor treatment outcomes [20,21].

Despite clinical recovery from MDD, anhedonia often persists as a residual symptom and contributes to recurrent episodes [22]. Existing treatments, including conventional antidepressants, such as selective serotonin reuptake inhibitors and serotonin-norepinephrine reuptake inhibitors, have shown limited efficacy in addressing anhedonia [23]. Similarly, psychotherapy offers only partial relief [24]. Current therapeutic approaches are primarily designed to alleviate depressive symptoms without adequately addressing the unique challenges posed by anhedonia [23,24]. As a result, anhedonia is closely linked to treatment-resistant depression, which is associated with increased healthcare costs and resource utilization [25]. Furthermore, the absence of evidence-based guidelines and approved medications for anhedonia exacerbates the difficulty in managing this condition [26].

While the prevalence of MDD in South Korea has been relatively well-documented, there remains a significant gap in understanding the prevalence and burden of anhedonia, as well as its implications for patients and economic outcomes. This study addresses this gap by investigating the prevalence and impact of anhedonia among MDD patients in South Korea. By examining the clinical and economic burden of anhedonia, this study aims to generate insights that can inform the development of targeted therapeutic strategies.

Moreover, the study explores discrepancies in treatment goals and satisfaction between patients and physicians, highlighting the misalignment that can hinder optimal care. The findings are expected to provide critical evidence for improving the management of anhedonia in patients with MDD and for fostering patient-centered approaches to treatment in South Korea.

## Materials and methods

### Study design and data source

This study was conducted as part of a large-scale, cross-sectional, observational study carried out across Australia, China, Japan, Malaysia, South Korea, and Taiwan during 18 April 2023–12 May 2023 [27,28]. Herein, this article reports findings specific to South Korea. Data were collected through an online self-reported survey conducted via a secure digital platform. Two separate survey instruments were employed: one targeting the general population (non-physicians) and another designed specifically for physicians. The general population-specific survey included structured items measuring sociodemographic characteristics, depression severity (9-item Patient Health Questionnaire (PHQ-9)), and anhedonia (14-item Snaith-Hamilton Pleasure Scale (SHAPS)). The physician-specific survey focused on clinical experience, patient caseload, perspectives on treatment goals and satisfaction. Both surveys utilized validated instruments and were pretested for cultural appropriateness in the South Korean context.

Only deidentified responses from all participants who completed the questionnaire and had provided informed consent online to participate were captured and analyzed. To ensure data completeness, the respondents were required to complete all questions in each section of the questionnaire before they could proceed to the next section. As a result of this survey design, there was no missing data.

The study adhered to the guidelines outlined in the Declaration of Helsinki and conformed to Good Epidemiological Practices as defined by the International Society for Pharmacoeconomics and Outcomes Research. The study protocol and questionnaire were submitted to Pearl Institutional Review Board (IRB) and received exemption status (IRB number: 023–0025). Throughout the study, strict confidentiality protocols were established to protect the security of all participants’ records and maintain anonymity. All respondents who participated in the study provided online informed consent and had the right to refuse and withdraw from the study.

Non-physician participants were recruited from opt-in databases representing the gender and age distributions of South Korea’s most recent countrywide census. Purposive sampling was used to select physicians from South Korea’s Health Insurance Review and Assessment (HIRA). Each respondent who passed the eligibility screener and provided informed consent was directed to complete a 30-minute survey (non-physician participants) or a 15-minute survey (physician participants).

### Study population

#### MDD patients.

Eligible participants aged ≥18 years, residents of South Korea, did not have a self-reported diagnosis of bipolar disorder or schizophrenia, and had provided informed consent were invited to participate in the study. All participants were asked to complete the PHQ-9 (score of ≥10) [29], and asked if they had a self-reported diagnosis of MDD. Participants who had scored ≥10 on the PHQ-9 [29] and had self-reported having a physician diagnosis of MDD were defined as patients with MDD and proceeded to self-complete SHAPS. Patients with MDD who had a SHAPS score of ≤2 [30,31] were defined as not having self-reported anhedonia (MDD non-ANH), while those who scored ≥3 on SHAPS scale [30,31] were defined as having self-reported anhedonia (MDD-ANH).

### Physician

The physician-specific survey was administered to physicians with ≥3 years of clinical experience as a practicing psychiatrist, who spent ≥30% of the time in direct patient care and treated ≥20 MDD patients in the past month.

### Sample size

A sample size of 350–400 respondents in the MDD group and 60 physicians was aimed per country, including South Korea. Based on an estimated 75% of patients experiencing anhedonia and a total of 290 participating physicians, the study was powered at 80% with a significance level of 0.05 to detect clinically relevant small to medium size differences (standardized difference between two means ranging from 0.2 to 0.5) between patients with MDD-ANH and MDD non-ANH, as well as patients with MDD-ANH and physicians.

### Study measures

#### Prevalence of MDD and MDD with anhedonia.

The prevalence of MDD was calculated as the total number of participants with a self-reported diagnosis of MDD who had a PHQ-9 score of ≥10 divided by the total number of eligible participants in the study. Similarly, the prevalence of MDD and anhedonia was calculated as the number of participants with a self-reported MDD diagnosis, a PHQ-9 score of ≥10, and a SHAPS score of ≥3 by the total number of eligible participants. All results were age- and gender-weighted using the United Nations (UN) estimations for South Korea to minimize potential sampling biases and provide higher representativeness to the national population [32].

### Participants characteristics

This study collected information on the characteristics (weighted) of patients with MDD-ANH and MDD non-ANH such as sociodemographic (age, sex, race or ethnicity, education, employment status, and household income) and health factors (body mass index [BMI], smoking or alcohol consumption habits, frequency of exercise, and diagnosis of comorbidities [by Charlson comorbidity index, CCI]). This study also measured weighted depression-specific characteristics for MDD-ANH vs MDD non-ANH such as time since depression diagnosis (in years), current prescription for depression, prior medication uses for depression, and current medication replaced or added to previous medication.

Physicians’ characteristics such as clinical experience (years) as a practicing psychiatrist, percentage of time spent in direct patient care, number of patients with MDD seen in the past month, and patient caseload (stratified by MDD-ANH and MDD non-ANH), were also recorded.

### Impact of anhedonia on MDD

The 14-item self-reported SHAPS was used to determine anhedonia in patients with MDD. It is a validated [33] and reliable scale that assesses a person’s incapacity to feel pleasure in a range of settings including social interactions, sensory stimulation (eating and drinking), hobbies, and interests [34,35]. A score of 1 was given if a respondent strongly disagreed or disagreed to a statement, and 0 was given if a respondent agreed or strongly agreed, summing up to a range of 0–14 [30]. The 7-Item Generalized Anxiety Disorder Assessment (GAD-7) scale was used to assess the severity of anxiety symptoms in the past 2 weeks. Response choices included “not at all”, “several days”, “more than half the days”, and “nearly every day.” The scores were summed to form a total that ranged from 0 to 21, with scores of 5, 10, and 15 indicating mild, moderate, and severe anxiety, respectively [36]. A 5-item Arizona Sexual Experience Scale (ASEX) was employed to quantify sexual functioning. These scores varied between 5 and 30, with higher scores indicating greater sexual dysfunction [37].

The RAND-36 HRQoL scale was used to assess the health-related quality of life [38]; two summary scores, physical component summary (PCS) and the mental component summary (MCS), were calculated. The scores ranged from 0 to 100, with higher scores denoting better HRQoL. The point difference of 3–5 between the groups was deemed to be a clinically important difference [39]. HRQoL was also measured using the 5-level EQ-5D questionnaire (EQ-5D-5L), which has an overall index score from 0 (health state equivalent to death) to 1 (perfect health) [40]. In addition, EQ-VAS was used by patients with MDD-ANH and MDD non-ANH to score their own health on a scale from “worst imaginable health state=0” to “best imaginable health state=100” [41]. Self-reported employment status was used to obtain data on labor force participation. Patients (MDD-ANH and MDD non-ANH) of working age (18–65 years) and employed full-time, part-time, self-employed, or students, were considered as participating in the labor force, while those who were not of working age and not employed but looking for work, not employed and not looking for work, on long-term disability, on short-term disability, retired, or a homemaker were considered as not participating in the labor force.

Work productivity was assessed using the WPAI (Work productivity and activity impairment questionnaire, a 6-item validated instrument), that comprises four parameters: presenteeism, absenteeism, overall work productivity loss (assessed in full-time, part-time, or self-employed patients with MDD-ANH and MDD non-ANH), and activity impairment (all patients with MDD-ANH and MDD non-ANH) [42].

The number of visits to general practitioners (GPs), psychiatrists, psychologists or therapists, and emergency rooms (ERs; “how many times have you been to the emergency room for your own medical condition in the past 6 months?”), as well as the frequency of hospitalizations (“how many times have you been hospitalized for your own medical condition in the past 6 months?”) were used to define healthcare resource utilization (HCRU).

### Patients and physicians’ perceived goals and satisfaction towards current depression therapeutics

All participants (MDD patients and physicians) rated the level of importance of treatment goals using a Likert scale ranging from 1 to 5 (1 = not at all important and 5 = extremely important) and expressed treatment satisfaction on a Likert scale ranging from 1 to 9 (1 = extremely dissatisfied, 9 = extremely satisfied); higher scores denoted greater treatment importance or satisfaction. MDD patients (regardless of anhedonia status) were asked to consider these with regard to their depression treatment. Physicians were asked to rate the importance of treatment goals for their patients with MDD-ANH. They were also asked to indicate their level of satisfaction with the performance of currently available pharmaceutical treatments for treating patients with MDD-ANH.

### Statistical analyses

Descriptive analyses were conducted on all study outcomes. Continuous and discrete variables were reported as mean±standard deviation (SD), whereas categorical variables were reported as frequencies and percentages. Chi-squared and *t-*tests were used to compare age- and gender-weighted PROs between MDD non-ANH and MDD-ANH patients. Multiple regression models were employed to examine the adjusted effect of anhedonia in the MDD sample, accounting for gender, age, BMI, smoking habits, alcohol consumption, frequency of exercise, education, employment, CCI and modified PHQ-9. To avoid collinearity, the anhedonia specific item of PHQ-9 (item 1) was removed. The remaining items of PHQ-9 were summed up and included in the regression analysis to adjust for depression severity. For exploratory purposes, all statistical analyses were performed using two-sided tests and results with p-values <0.05 were considered statistically significant. Data were analyzed using SPSS version 29 (IBM), R version 4.2.2, and SAS version 9.4.

## Results

### Prevalence of MDD and MDD with anhedonia

Of the 4496 participants, 1112 (24.7%) self-reported a PHQ-9 score of ≥10. The age- and gender-weighted prevalence of MDD with self-reported PHQ-9 score of ≥10 and a physician diagnosis was 9.9% (n = 447); while 6.1% (n = 275) had anhedonia. Age- and gender-weighted prevalence of anhedonia among MDD patients was 61.5% (n = 275); the rates of depression severity (moderate n = 130, 47.3%; severe n = 48, 17.5%) among overall anhedonia patient population were similar to the overall MDD patient population (Table 1). There was a low correlation (r = 0.08, p = 0.093) between PHQ-9 item 1 and SHAPS score (S1 Table).

**Table 1:** Age- and- gender weighted PHQ-9 and SHAPS scores among the total survey sample.

Variables	Total Survey population (N = 4496)	Total MDD patients (n = 447)	Total MDD patients with anhedonia (n = 275)
	n (%)	n (%)	n (%)
**PHQ-9**			
Below threshold (PHQ-9 < 10)	3385 (75.3)	0 (0.0)	0 (0.0)
Moderate (10–14)	648 (14.4)	218 (48.8)	130 (47.3)
Moderate to Severe (15–19)	317 (7.1)	137 (30.6)	97 (35.3)
Severe (20–27)	147 (3.3)	92 (20.6)	48 (17.5)
Moderate Depression or greater (PHQ-9 ≥ 10)	1112 (24.7)	–	–
**Presence of MDD** ^**a**^	447 (9.9)	–	–
**SHAPS** ^**b**^			
0	56 (1.2)	56 (12.5)	0 (0.0)
1-2	65 (1.4)	65 (14.5)	0 (0.0)
Presence of Anhedonia (≥3)	275 (6.1)	275 (61.5)	275 (100.0)

Note: Prevalence rates were weighted based on age and gender weighted using the UN population estimates for South Korea.

^a^Respondents are classified under MDD if they reported moderate depression or greater (PHQ-9 ≥ 10) and a self-reported physician diagnosis of depression.

^b^SHAPS is surveyed among respondents with moderate depression or greater (PHQ-9 ≥ 10) and a self-reported physician diagnosis of depression.

ANH, anhedonia; MDD, major depressive disorder; PHQ-9, 9-item patient health questionnaire; SHAPS, Snaith-Hamilton pleasure scale; UN, United Nations.

### Patient characteristics

**Fig 1:**
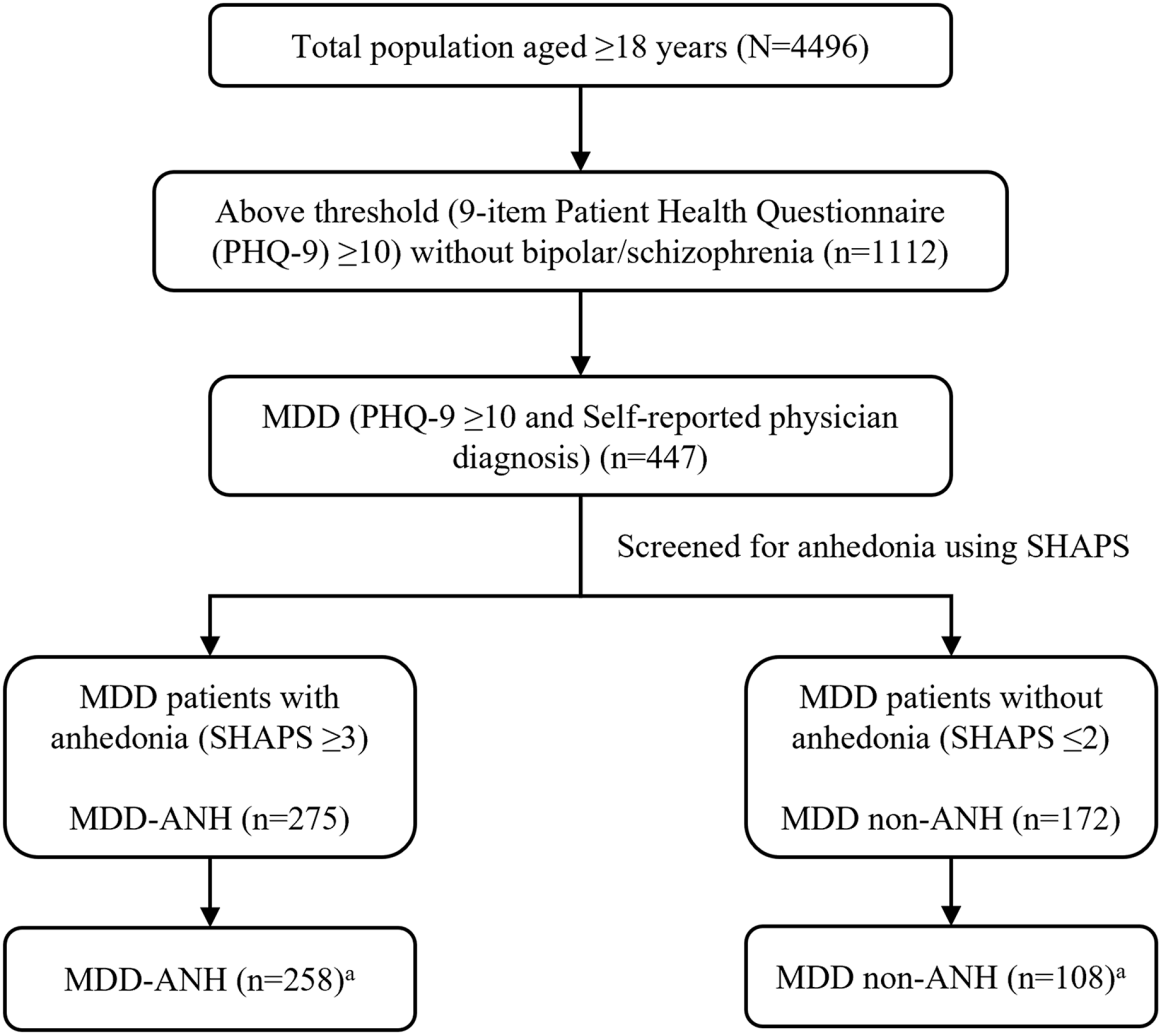
Study Population. ^a^MDD patients who completed all survey questions and included in analysis. ANH, anhedonia; MDD, major depressive disorder; MDD-ANH, MDD with anhedonia; MDD non-ANH, MDD without anhedonia; PHQ-9, 9-item patient health questionnaire; SHAPS, Snaith-Hamilton pleasure scale.

Overall, 258 patients with MDD-ANH and 108 with MDD non-ANH completed the survey (**Fig 1**). Half of the MDD-ANH group were female (50.4%), whereas the percentage of female patients was numerically lower in the MDD non-ANH group (44.9%). The mean age of patients with MDD-ANH and MDD non-ANH was 38.36 and 40.09, respectively. Most patients in both groups went to college (MDD-ANH: 60.1%; MDD non-ANH: 63.4%). The employment status was similar in both groups, with a higher proportion of retirees in the MDD-ANH group versus the MDD non-ANH group (5.7% vs. 0.0%; **Table 2**).

**Table 2:** Demographics and patient characteristics (weighted) of MDD-ANH and MDD non-ANH population.

Variables	MDD-ANH (n = 258)	MDD non-ANH (n = 108)
Sociodemographic characteristics		
Gender, n (%)		
Male	128 (49.6)	59 (55.2)
Female	130 (50.4)	48 (44.9)
Age, years, mean (SD)	38.36 (16.06)	40.09 (13.67)
Age Category, years, n (%)		
18 to <25	56 (21.7)	12 (11.3)
25 to <35	72 (27.8)	33 (30.6)
35 to <45	66 (25.5)	32 (29.3)
45 to <55	33 (12.8)	19 (18.0)
55 to <65	14 (5.4)	4 (3.4)
65 and older	18 (6.9)	8 (7.4)
Education, n (%)		
Elementary school	1 (0.4)	0 (0.0)
Junior high school	3 (1.2)	1 (0.9)
High school	57 (21.9)	20 (18.7)
2-year college	30 (11.6)	10 (9.0)
College	155 (60.1)	68 (63.4)
Graduate school	13 (4.9)	6 (5.8)
No school	0 (0.0)	2 (2.3)
Employment status, n (%)		
Employed full time	143 (55.5)	70 (64.6)
Self-employed	19 (7.4)	14 (12.6)
Employed part time	28 (10.9)	12 (11.0)
Homemaker	18 (7.0)	4 (4.1)
Retired	15 (5.7)	0 (0.0)
Student	15 (5.9)	2 (2.3)
Not employed, but looking for work	18 (6.9)	4 (3.9)
Not employed and not looking for work	7 (2.8)	3 (2.4)
General health characteristics		
BMI, mean (SD)	22.29 (4.16)	22.09 (3.41)
Frequency of smoking, n (%)		
Everyday	126 (48.7)	49 (45.2)
Some days	30 (11.8)	37 (33.9)
Not at all	102 (39.6)	23 (20.9)
Frequency of alcohol consumption, n (%)		
Every day	70 (27.1)	27 (25.4)
Some days	151 (58.7)	72 (67.1)
Not at all	37 (14.2)	8 (7.5)
Frequency of weekly exercise, n (%)		
More than 5 times a week	35 (13.7)	28 (26.4)
3-5 times a week	69 (26.8)	33 (30.3)
1-2 times a week	80 (31.1)	32 (29.9)
Very rarely or never	73 (28.4)	14 (13.4)
CCI, mean (SD)	1.51 (2.54)	2.00 (3.07)
CCI score categories, n (%)		
0	144 (55.7)	56 (51.5)
1	33 (12.9)	10 (9.3)
2	21 (8.0)	14 (12.6)
3+	60 (23.4)	29 (26.6)

Note: Prevalence rates were weighted based on age and gender weighted using the UN population estimates for South Korea.

ANH, anhedonia; BMI, body mass index; MDD, major depressive disorder; MDD-ANH, MDD with anhedonia; MDD non-ANH, MDD without anhedonia; CCI, Charlson comorbidity index; SD, standard deviation; UN, United Nations.

The distribution of patients smoking frequency was different between the MDD-ANH group and the MDD non-ANH group. A higher proportion of patients with MDD-ANH very rarely or never exercised than MDD non-ANH patients (28.4% vs. 13.4%). The mean CCI scores were numerically lower in the MDD-ANH group (1.51), compared with the MDD non-ANH group (2.00; **Table 2**). The unweighted characteristics of MDD patients are presented in S2 Table.

### Physician characteristics

A total of 60 physicians completed the survey. The physicians had a mean clinical experience of 10.82 years practicing as psychiatrists and had spent around 81.8% of their time in direct patient care. The mean caseload was 197.68 patients with MDD in the past month, of which, physicians had perceived 37.5% of their MDD patients in their clinical settings to have anhedonia (S3 Table).

### Clinical characteristics of patients with MDD

The mean duration of MDD since diagnosis was significantly longer in patients with MDD-ANH compared with MDD non-ANH (7.71 vs. 5.74 years; p = 0.0206). The proportion of patients currently taking antidepressants was significantly lower in the MDD-ANH group compared with the MDD non-ANH group (47.6% vs. 66.8%; p = 0.0032). Prior depression medication use was significantly lower in patients with MDD-ANH than MDD non-ANH (55.3% vs. 72.0%; p = 0.0466). In patients with MDD-ANH versus MDD-non-ANH, current medication was replaced by existing medication in 35.1% versus 59.5% (p = 0.0001), while current medication was added to the existing medication in 53.1% versus 40.5% (p = 0.0001). A significantly higher proportion of patients with MDD-ANH (43.3%) than MDD non-ANH (6.1%) have been recommended treatment previously but are not currently on medication (p = 0.0024; **Table 3**).

**Table 3:** Depression-specific characteristics (weighted) of MDD-ANH versus MDD non-ANH population.

Variable	MDD-ANH (n = 258)	MDD non-ANH (n = 108)	p-value
Time since depression diagnosis, years, mean (SD)	7.71 (10.89)	5.74 (5.25)	0.0206
Current prescription use for depression, n (%)	123.0 (47.6)	72.0 (66.8)	0.0032
Prior depression medication use^a^, n (%)	68.0 (55.3)	52.0 (72.0)	0.0466
Current medication replaced or added to previous medication, n (%)			
Replaced my existing medication, treatment, or therapy	24.0 (35.1)	31.0 (59.5)	0.0001
Added on to my existing medication, treatment or therapy	36.0 (53.1)	21.0 (40.5)	
Not sure	8.0 (11.8)	0.0 (0.0)	
Doctor ever recommended a prescription to treat depression, n (%)	29 (43.3)	1 (6.1)	0.0024

^a^Asked of those reporting having experienced depression in the past 12 months who are taking a prescription medication.

ANH, anhedonia; MDD, major depressive disorder; MDD-ANH, MDD with anhedonia; MDD non-ANH, MDD without anhedonia; SD, standard deviation.

### Impact of anhedonia on MDD

#### Health-related quality of life (HRQoL).

The MDD-ANH group had significantly worse HRQoL scores compared with the MDD non-ANH group, with β-coefficient −6.41 (95% confidence interval [CI]: −10.60, −2.22; p = 0.003) for EQ-VAS. Patients with MDD-ANH scored worse in other HRQoL measures, albeit not statistically significant: −0.14 (−1.68, 1.41) for RAND-PHC (physical health component) and −1.04 (−2.46, 0.39) for RAND-MHC (mental health component), and −0.01 (−0.04, 0.03) for EQ-5D. Patients with MDD-ANH had higher odds of experiencing sexual dysfunction compared with patients with MDD non-ANH (odds ratio [OR]=1.85, 95% CI: 1.17, 2.93; p = 0.009; **Table 4**).

**Table 4:** Multivariable regression analysis of patient-centric, economic, and clinical outcomes in MDD-ANH versus MDD non-ANH.

Outcomes	MDD-ANH (adjusted mean)	MDD non-ANH (adjusted mean)	β-coefficient, odds ratio or, rate ratio (95% CI)	p-value^a^
**Sexual functioning** ^**b,c**^	**n = 260**	**n = 119**	**Odds ratio**	
Arizona sexual health scale	0.51	0.36	1.85 (1.17, 2.93)	0.009
**HRQoL** ^**d**^	**n = 260**	**n = 119**	**Beta-coefficient**	
RAND – MHC	28.29	29.32	−1.04 (−2.46, 0.39)	0.155
RAND – PHC	34.73	34.87	−0.14 (−1.68, 1.41)	0.863
EQ-5D Index score	0.68	0.69	−0.01 (−0.04, 0.03)	0.712
EQ-VAS score	51.93	58.34	−6.41 (−10.60, −2.22)	0.003
**Mental health** ^**d**^	**n = 260**	**n = 119**	**Beta-coefficient**	
GAD-7 score	11.70	12.13	−0.43 (−1.10, 0.24)	0.211
**Labor force participation** ^**b,c**^	**n = 257**	**n = 118**	**Odds ratio**	
Participating in the labor force	0.88	0.90	0.83 (0.40, 1.73)	0.611
**WPAI** ^**e,c**^			**Rate ratio**	
Absenteeism	**n = 217**	**n = 105**	0.73 (0.54, 0.99)	0.042
	14.43	19.69		
Presenteeism	**n = 217**	**n = 105**	0.98 (0.89, 1.08)	0.672
	57.09	58.25		
Overall Work Productivity Impairment	**n = 217**	**n = 105**	0.97 (0.88, 1.06)	0.473
	62.36	64.45		
Activity Impairment	**n = 260**	**n = 119**	1.01 (0.92, 1.10)	0.916
	55.71	55.43		
**HCRU** ^**e,c**^	**n = 260**	**n = 119**	**Rate Ratio**	
Number of GP visits in past 6 months	0.25	0.08	3.09 (1.03, 9.30)	0.040
Number of ER visits in past 6 months	0.81	1.79	0.45 (0.18, 1.15)	0.095
Number of Hospitalizations in past 6 months	0.31	0.72	0.43 (0.26, 0.71)	0.001
Number of Psychiatrist visits in past 6 months	0.87	0.95	0.91 (0.48, 1.72)	0.772
Number of Psychologist/therapist visits in past 6 months	0.33	0.25	1.31 (0.59, 2.93)	0.508

^a^Note: *P* value was calculated based on the comparison of MDD-ANH versus MDD non-ANH. controlling for race, sex, age, BMI, frequency of smoking, frequency of consuming alcohol, frequency of exercise, education, employment status, CCI, and PHQ-9 (Item 1 removed)

^b^Binary logistic regression model. Results are reported as odds ratios.

^c^Covariates restricted to age, sex, CCI, and PHQ-9 (Item 1 removed) due to convergence issues.

^d^GLM with normal distribution and identity link. Results are reported as beta coefficients.

^e^GLM with negative binomial distribution and log-link. Results are reported as rate ratios.

ANH, anhedonia; CCI, Charlson comorbidity index; CI, confidence interval; EQ-5D, EuroQol 5-dimension health questionnaire; ER, emergency room; GAD-7, 7-item generalized anxiety disorder assessment scale; GP, general physician; HCRU, healthcare resource utilization; HRQoL, health-related quality of life; MDD, major depressive disorder; MDD-ANH, MDD with anhedonia; MDD non-ANH, MDD without anhedonia; MHC: mental health composite; PHC- physical health composite; VAS, visual analogue scale; WPAI, work productivity and activity impairment.

### Work productivity and activity impairment (WPAI)

Multivariate analysis showed that patients in the MDD-ANH group, compared with the MDD non-ANH group, had significantly lower rates of absenteeism after adjusting for age, sex, CCI, and PHQ-9 (item 1 removed), rate ratio (RR)=0.73 (95% CI: 0.54 to 0.99; p = 0.0423). MDD-ANH respondents reported lower rates of labor force participation, but those were not statistically significant (OR=0.83, 95% CI: 0.40, 1.73; p = 0.611) (**Table 4**).

### Healthcare resource utilization (HCRU)

Multivariate analyses showed that patients in the MDD-ANH group had more GP visits (RR [95% CI] 3.09 [1.03, 9.30]; p = 0.04) but fewer hospitalizations in the past 6 months (RR [95% CI] 0.43 [0.26, 0.71]; p = 0.001), compared with those in the MDD non-ANH group (**Table 4**). A numerically higher proportion of patients in the MDD-ANH group, compared with the MDD non-ANH group, visited psychiatrists (22.3% vs. 20.7%; p = 0.7499) or psychologists/therapists (17.5% vs. 8.7%; p = 0.0512; S4 Table). Detailed HCRU data is presented in S4 Table.

### Patient and physician perceived treatment goals and satisfaction towards current therapeutics for anhedonia

The level of importance of perceived treatment goals varied among patients with MDD-ANH and MDD non-ANH, and physicians, as described in **Table 5**. The top ranked perceived treatment goals by patients with MDD-ANH included improving sleep quality, regaining self-esteem, and having positive emotions. However, there was notable discordance with physicians’ ranked treatment goals, who prioritized avoiding suicidal thoughts, controlling depressed mood, and improving sleep quality (**Table 5**).

**Table 5:** Treatment expectations (goals) and satisfaction in treating MDD with anhedonia among MDD-ANH, MDD non-ANH, and physicians.

Variables	MDD-ANH patients		MDD non-ANH patients		Physicians		p-value	
	Ranking	mean (SD)	Ranking	mean (SD)	Ranking	mean (SD)	MDD-ANH vs. Physicians	MDD non-ANH vs. Physicians
**Level of importance of treatment goals (scale 1–5)** ^**a**^								
Improve sleep quality	**1**	3.91 (1.00)	**3**	3.77 (1.02)	**3**	4.02 (0.89)	0.4302	0.114
Regain self-esteem	**2**	3.74 (1.04)	**4**	3.75 (1.06)	6	3.77 (0.72)	0.8409	0.8914
Have positive emotions (e.g., optimism)	**3**	3.69 (1.03)	9	3.53 (0.97)	9	3.67 (0.84)	0.8844	0.3809
Control depressed mood (e.g., sadness)	**4**	3.64 (1.10)	**2**	3.82 (0.92)	**2**	4.03 (0.61)	0.0002	0.0747
Reduce psychological anxiety (e.g., feeling irritable and worried)	**5**	3.63 (1.02)	7	3.60 (0.97)	8	3.70 (0.91)	0.6376	0.5045
Avoid having suicidal thoughts	6	3.60 (1.28)	**1**	3.92 (1.01)	**1**	4.28 (0.76)	<.0001	0.0103
Reduce the feeling of fatigue	7	3.54 (1.00)	**5**	3.69 (0.93)	7	3.72 (0.72)	0.1229	0.8598
Restore normal social function (i.e., participate and enjoy relationships with family, and friends)	8	3.47 (0.95)	6	3.67 (1.08)	**5**	3.83 (0.83)	0.0072	0.2795
Improve productivity at work, school, and home (e.g., household chores)	9	3.43 (1.03)	8	3.54 (1.07)	**4**	3.85 (0.80)	0.0008	0.0344
Regain interest in hobbies	10	3.41 (1.06)	11	3.38 (0.84)	9	3.67 (0.82)	0.0412	0.036
Increase attention span	11	3.39 (1.06)	10	3.43 (0.96)	10	3.65 (0.82)	0.0408	0.1299
Regain appetite	12	2.97 (1.10)	12	3.35 (0.97)	9	3.67 (0.73)	<.0001	0.0194
Improve sexual satisfaction	13	2.75 (1.22)	13	3.31 (1.06)	11	3.27 (0.80)	0.0001	0.7823
**Level of satisfaction with treatment goals (scale 1–9)** ^**b**^								
Control depressed mood (e.g., sadness)	**1**	5.66 (1.84)	**4**	6.18 (1.64)	**3**	6.78 (1.34)	<.0001	0.0185
Have positive emotions	**2**	5.59 (1.91)	**2**	6.24 (1.38)	11	5.65 (1.57)	0.8178	0.0142
Avoid having suicidal thoughts	**3**	5.58 (1.98)	**3**	6.22 (1.59)	6	6.17 (1.63)	0.0367	0.836
Regain appetite	**4**	5.52 (1.77)	9	5.99 (1.65)	**4**	6.73 (1.22)	<.0001	0.0014
Improve sleep quality	**5**	5.51 (1.92)	**1**	6.28 (1.70)	**1**	7.15 (1.39)	<.0001	0.001
Improve productivity at work, school, and home (e.g., household chores)	6	5.42 (1.75)	**4**	6.18 (1.64)	**5**	6.28 (1.52)	0.0007	0.6834
Restore normal social function (i.e., participate and enjoy relationships with family, and friends)	7	5.41 (1.96)	**5**	6.17 (1.65)	7	5.87 (1.68)	0.1051	0.2692
Reduce psychological anxiety (e.g., feeling irritable and worried)	8	5.33 (1.99)	6	6.15 (1.58)	**2**	7.07 (1.16)	<.0001	0.0001
Increase attention span	9	5.22 (1.80)	10	5.97 (1.72)	8	5.85 (1.53)	0.0154	0.6641
Regain interest in hobbies	10	5.20 (2.02)	8	6.02 (1.71)	10	5.70 (1.64)	0.0846	0.2504
Regain self-esteem	11	5.16 (1.89)	11	5.95 (1.69)	12	5.12 (1.78)	0.8661	0.0039
Reduce the feeling of fatigue	12	5.04 (1.97)	7	6.14 (1.63)	9	5.78 (1.62)	0.0082	0.1844
Improve sexual satisfaction	13	5.02 (1.96)	12	5.86 (1.62)	13	4.85 (1.80)	0.552	0.0004

^a^Likert scale ranging from 1 to 5 (1-not at all important, 5-extremely important).

^b^Likert scale ranging from 1 to 9 (1-extremely dissatisfied, 9-extremely satisfied).

ANH, anhedonia; MDD, major depressive disorder; MDD-ANH, MDD with anhedonia; MDD non-ANH, MDD without anhedonia; SD, standard deviation.

Compared to MDD-ANH patients, physicians had placed significantly greater importance on treatment goals in terms of controlling depressed mood (p = 0.0002), regaining appetite (p < 0.0001), regaining interest in hobbies (p = 0.0412), avoiding suicidal thoughts (p < 0.0001), improving productivity at work, school, and home (p = 0.0008), restoring normal social function (p = 0.0072), improving sexual satisfaction (p = 0.0001), and increasing attention span (p = 0.0408; **Table 5**).

The perceived level of treatment satisfaction towards treatment goals ranged between 5.02–5.66 in the MDD-ANH group, 5.86–6.28 in the MDD non-ANH group, and 4.85–7.15 among physicians. Patients with MDD-ANH ranked the highest level of satisfaction with treatment goals of controlled depressed mood, having positive emotions, and avoiding suicidal thoughts. On the contrary, physicians ranked a higher level of satisfaction with improving sleep quality, reducing psychological anxiety, and controlling depressed mood.

Physicians had reported significantly higher levels of satisfaction than patients with MDD-ANH in 8 of 13 treatment goals. Patients with MDD-ANH had significantly lower treatment satisfaction for controlling depressed mood, regaining appetite, improving sleep quality, improving productivity at work, school, and home, and reducing psychological anxiety (all, p < 0.001) compared to physicians. Common treatment goals with the lowest levels of satisfaction among patients with MDD-ANH and physicians included regaining interest in hobbies, regaining self-esteem, and improving sexual satisfaction (**Table 5**).

## Discussion

Present study was part of a large-scale cross-sectional analysis conducted across Australia, China, Japan, Malaysia, Taiwan, and South Korea. The results on the impact of anhedonia among patients with MDD in Asia-pacific region were published earlier [27,28]. Here, we present data on the prevalence of anhedonia among patients with MDD and the impact of anhedonia on the disease burden associated with MDD particularly in South Korea. The study also investigated patients’ and physicians’ expectations and perceived satisfaction towards pharmaceutical treatments for anhedonia in patients with MDD. To the best of our knowledge, this is the first report of such data from South Korea.

In this study, 61.5% of patients with MDD exhibited symptoms of anhedonia, as estimated by their self-reported SHAPS score, which falls within the prevalence rates observed in the previous studies (35–75%) [43–46]. This indicates that anhedonia is a core feature of MDD, and its high prevalence warrants the necessity for diagnosis, treatment and understanding of the disease burden. Notably, the prevalence of anhedonia among patients with MDD perceived by physicians in a clinical setting was substantially lower than patients’ self-reported anhedonia measured by SHAPS ≥3. Even though anhedonia is a core symptom in MDD, physicians may prioritize treating depressive symptoms, such as depressed mood over anhedonia. This may arise from a limited understanding of anhedonia due to lack of consensus on definition and assessment of anhedonia in MDD among the medical community. Physicians may also be more attuned to and ready to address other more familiar symptoms associated with MDD. Furthermore, patients’ inability to communicate symptoms associated with anhedonia to their physicians, potentially due to challenges in describing their symptoms or a lack of awareness of the significance of such symptoms, may also affect the diagnosis and management of anhedonia by physicians [45]. Therefore, addressing these challenges would require improved patient-physician communication regarding anhedonia. Using validated anhedonia assessment tools such as SHAPS could enhance the accuracy of diagnosis and guide treatment decisions and improve the clinical management of anhedonia in MDD.

Patients with MDD-ANH reported a significantly longer duration of depression since diagnosis compared to patients with MDD non-ANH, which may be associated with chronic depression or reflect the persistence of anhedonia despite treatment [23,46,47]. This finding suggests that anhedonia may contribute to the chronicity and persistence of depressive symptoms. Therefore, long-term management plans should be developed, focusing not only on immediate symptom relief but also on the enduring improvement of patients’ HRQoL and functional outcomes.

In addition, MDD-ANH had a lower current prescription use compared to MDD non-ANH group, suggesting a gap in current therapeutics for anhedonia symptom management and the need for innovative treatments. Furthermore, the proportion of patients with prior depression medication use was lower in the MDD-ANH group than in the MDD non-ANH group. This may be linked to the lack of targeted therapy approved for the treatment of anhedonia in patients with MDD or may suggest undertreatment owing to lack of persistence to treatment [48].

Moreover, a lower proportion of patients in the MDD-ANH group replaced their current medication while a higher proportion of patients added on to the existing treatment, compared with the MDD non-ANH group. This could be attributed to anhedonia being associated with treatment-resistant depression and is an indicator for prolonged recovery periods [20,49]. Additionally, the Korean Medication algorithm for depressive disorder suggests adding augmenting drugs in case of an inadequate treatment response, which could also explain the above finding, as physicians might be adhering to current treatment guidelines on depression [50]. The lower probability of drug replacement and the higher probability of receiving additional prescription may reflect physician’s attempts to control anhedonia symptoms by adding on various drugs to the existing treatment, which could be associated with increased risk of drug induced complications and healthcare cost. Owing to the cross-sectional nature of this study, no direct conclusion can be made. However, these suboptimal treatment regimens may be associated with a prolonged disease burden in patients with MDD-ANH, highlighting an unmet need for new drugs targeting anhedonia.

The study assessed the impact of anhedonia on HRQoL, work productivity and HCRU. Previous studies have reported a significant impact of anhedonia on HRQoL in patients with depression. A longitudinal study reported reduced HRQoL, and poorer life enjoyment and satisfaction (social and family relationships, daily functioning, sexual interest, overall well-being etc.) in patients with mood disorders (unipolar and bipolar) and anhedonia compared with healthy adults [51]. Similarly, a cross-sectional survey at a general medical outpatient unit in São Paulo in patients with severe medical conditions reported significant inverse association between anhedonia and HRQoL [52]. Reinforcing these results, a recent meta-analysis also reported reduced HRQoL and functional outcomes with anhedonia [53]. Aligning with the previous studies, the results of the present study demonstrated significantly worse HRQoL in patients with MDD-ANH compared to patients with MDD non-ANH, as indicated by the EQ VAS score. The results also showed worsening sexual dysfunction in patients with MDD-ANH compared to patients with MDD non-ANH. This is in line with previous studies showing an association of anhedonia with sexual dysfunction [54,55]. Collectively, the findings highlight a need to prioritize assessment and treatment of anhedonia in patients with MDD.

Anhedonia in MDD also impair the willingness to expend efforts for rewards, wherein MDD patients with high anhedonia exhibited neurobiological deficits in mechanisms underlying reward liking, reward wanting and reward learning [56,57]. This may be related to the lower labor force participation observed in patients with MDD-ANH, as these patients may not perceive incentive motivation associated with labor force participation. This suggests a need for targeted interventions that address these underlying neurobiological deficits and enhance reward processing. The hospitalization frequency was low in both patients with MDD-ANH and patients with MDD non-ANH. However, further research is needed to understand the reasons underlying this observation, i.e., availability of outpatient care services to manage acute symptoms.

In the current study, a discordance in treatment goals and in treatment satisfaction was observed between patients with MDD-ANH and physicians, suggesting that physicians may not adequately align with the key concerns of patients with MDD-ANH. Patients with MDD-ANH prioritized treatment goals such as improving sleep quality, regaining self-esteem, and having positive emotions, contrary to physicians who rated avoiding suicidal thoughts, controlling depressed mood and improving sleep quality mood as their top priorities.

In the majority of treatment goals, the level of treatment satisfaction rated by patients with MDD-ANH was lower than that rated by physicians such as controlling depressed mood, regaining appetite, improve sleep quality, improving productivity at work, school, and home, and reducing psychological anxiety, suggesting a potential misalignment between physicians and patients in the perception of treatment success, regardless of anhedonia. On the contrary, closer alignment in treatment expectations and satisfaction was observed between patients with MDD non-ANH and physicians. Both groups rated avoiding suicidal thoughts, controlling mood, and improving sleep quality as the top three most important treatment goals, with improving sleep quality having received the highest satisfaction. This potentially suggests that treatment expectations and satisfaction among physicians are more closely aligned with patients with MDD non-ANH, which further insinuates a gap in addressing the treatment needs of patients with MDD-ANH. Therefore, there is a need for regular assessment of self-reported outcomes of patients with MDD-ANH, including but not limited to PHQ-9, to get a better understanding of the patient’s perspective. Shared decision making between patients and physicians may help in aligning treatment goals and understanding patient perspectives [58,59].

Moreover, patients with MDD-ANH reported the lowest satisfaction for treatment goal of improving sexual satisfaction, which is rather a characteristic symptom of anhedonia. On the other hand, improved sleep quality was reported as the most prioritized treatment goal among patients with MDD. This could be attributed to the reluctance of patients in South Korea to discuss sensitive topics such as sexual satisfaction with physicians, owing to sociocultural beliefs and lack of awareness [60,61]. This highlights the unmet clinical need in patients with MDD-ANH, emphasizing the importance of candid communication to improve the overall treatment outcomes.

Furthermore, these findings suggest that once the prioritized core symptoms of MDD are resolved, it is important to assess and resolve residual symptoms including symptoms related to anhedonia, such as improving sexual satisfaction. Sexual dysfunction has been associated with reduced HRQoL and overall well-being in patients with MDD [62]. Additionally, conventional antidepressants can worsen sexual function, leading to reduced treatment adherence [63]. Therefore, timely recognition of sexual dysfunction allows for the use of appropriate intervention, thereby improving the overall HRQoL of patients with MDD [62,63].

Overall, anhedonia was associated with increased disease burden in terms of reduced HRQoL and increased HCRU, and a lower level of treatment satisfaction towards perceived treatment goals. The findings highlight the need for novel therapeutics to treat anhedonia and realistic tools to address the disparities in treatment goals and effective evaluation between the patients and their treating physicians.

This study provides valuable insights into the significant burden of anhedonia among patients with MDD in South Korea, emphasizing its impact on HRQoL, HCRU, and treatment satisfaction. However, several limitations should be noted.

First, the cross-sectional design limits the ability to establish causal relationships between anhedonia and the outcomes assessed. Longitudinal studies are needed to explore the progression and long-term impact of anhedonia. Second, the reliance on self-reported data introduces potential biases, such as recall and social desirability bias, which may affect the accuracy of sensitive information like suicidal ideation. Incorporating clinical-reported assessments could address these issues. Third, given the exploratory nature of this study and the number of variables analyzed, there is a possibility of Type I error. Therefore, the findings should be interpreted with caution. Additionally, the use of an online survey may have excluded certain demographic groups, potentially limiting the generalizability of the findings. Future studies should include more diverse sampling methods to ensure broader representation. Lastly, no formal insights or cognitive capacity assessment were performed during the recruitment process and individuals with more severe mental conditions or cognitive impairment may be underrepresented. This limits the generalizability of the study findings to the wider spectrum of MDD patients.

Despite these limitations, the study contributes critical evidence to guide clinical and policy efforts in improving care for MDD patients with anhedonia.

## Conclusions

This study is the first to comprehensively investigate the prevalence and burden of anhedonia among patients with MDD in South Korea. The findings provide critical insights into the significant impact of anhedonia on HRQoL, HCRU, and treatment satisfaction. Patients with MDD-ANH exhibited greater disease burden compared to those without anhedonia, emphasizing the urgent need for more targeted and effective management strategies.

Additionally, this study highlights key discrepancies between patients’ and physicians’ treatment goals, underscoring the importance of improved patient-physician communication and incorporation of shared decision-making into clinical practice. These results point to the necessity of routine assessment of anhedonia using validated tools, such as SHAPS, to better identify unmet needs and guide personalized treatment approaches.

As the first study of its kind in South Korea, these findings contribute to a deeper understanding of the unique challenges faced by MDD patients in this context. By addressing the burden of anhedonia, integrating focused interventions, and aligning treatment priorities between patients and physicians, this study lays the groundwork for advancing patient-centered care and improving mental health outcomes in South Korea.

## Supporting information

S1 Table Weighted correlation between SHAPS and PHQ-9 scores. (PDF)

S2 Table Patient’s characteristics (unweighted) of MDD-ANH and MDD non-ANH population. (PDF)

S3 Table Physicians’ clinical experience and perceived patient load with MDD. (PDF)

S4 Table Weighted healthcare resource utilization in MDD-ANH versus MDD non-ANH population. (PDF)

## References

[1] Global Health Metrics. Major depressive disorder — Level 4 cause. Global Health Metrics. https://www.healthdata.org/results/gbd_summaries/2019/major-depressive-disorder-level-4-cause 2019.

[2] Cho MJ, Kim J-K, Jeon HJ, Suh T, Chung I-W, Hong JP, et al. Lifetime and 12-month prevalence of DSM-IV psychiatric disorders among Korean adults. J Nerv Ment Dis. 2007;195(3):203–10. doi: 10.1097/01.nmd.0000243826.40732.45 17468679

[3] Cho MJ, Chang SM, Lee YM, Bae A, Ahn JH, Son J, et al. Prevalence of DSM-IV major mental disorders among Korean adults: A 2006 National Epidemiologic Survey (KECA-R). Asian J Psychiatr. 2010;3(1):26–30. doi: 10.1016/j.ajp.2010.01.009 23051134

[4] Cho MJ, Seong SJ, Park JE, Chung I-W, Lee YM, Bae A, et al. Prevalence and Correlates of DSM-IV Mental Disorders in South Korean Adults: The Korean Epidemiologic Catchment Area Study 2011. Psychiatry Investig. 2015;12(2):164–70. doi: 10.4306/pi.2015.12.2.164 25866515 PMC4390585

[5] Kim GE, Jo M-W, Shin Y-W. Increased prevalence of depression in South Korea from 2002 to 2013. Sci Rep. 2020;10(1):16979. doi: 10.1038/s41598-020-74119-4 33046758 PMC7550589

[6] Lee J, Kim H, Hong JP, Cho S-J, Lee J-Y, Jeon HJ, et al. Trends in the Prevalence of Major Depressive Disorder by Sociodemographic Factors in Korea: Results from Nationwide General Population Surveys in 2001, 2006, and 2011. J Korean Med Sci. 2021;36(39):e244. doi: 10.3346/jkms.2021.36.e244 34636501 PMC8506416

[7] Tackling the mental health impact of the COVID-19 crisis: An integrated, whole-of-society response. Organisation for Economic Co-Operation and Development (OECD). 2021. doi: 10.1787/0ccafa0b-en

[8] Lee H-S, Dean D, Baxter T, Griffith T, Park S. Deterioration of mental health despite successful control of the COVID-19 pandemic in South Korea. Psychiatry Res. 2021;295:113570. doi: 10.1016/j.psychres.2020.113570 33234326 PMC7664364

[9] Health at a Glance 2023. Health at a Glance. OECD. 2023. doi: 10.1787/7a7afb35-en

[10] Lee J, Ko Y-H, Shin C, Han R, Chae N, Yoon H-K. Suicide and Suicide Prevention Awareness in Korea During the COVID-19 Pandemic. Psychiatry Investig. 2022;19(10):847–56. doi: 10.30773/pi.2022.0108 36327965 PMC9633166

[11] American Psychiatric Association. Diagnostic and statistical manual of mental disorders: DSM-5. 5th ed. Washington, D.C.: American Psychiatric Publishing. 2013.

[12] Der-Avakian A, Markou A. The neurobiology of anhedonia and other reward-related deficits. Trends Neurosci. 2012;35(1):68–77. doi: 10.1016/j.tins.2011.11.005 22177980 PMC3253139

[13] Birnie MT, Levis SC, Mahler SV, Baram TZ. Developmental Trajectories of Anhedonia in Preclinical Models. Curr Top Behav Neurosci. 2022;58:23–41. doi: 10.1007/7854_2021_299 35156184 PMC10226722

[14] Zimmerman M, Ellison W, Young D, Chelminski I, Dalrymple K. How many different ways do patients meet the diagnostic criteria for major depressive disorder?. Compr Psychiatry. 2015;56:29–34. doi: 10.1016/j.comppsych.2014.09.007 25266848

[15] Gong L, Yin Y, He C, Ye Q, Bai F, Yuan Y, et al. Disrupted reward circuits is associated with cognitive deficits and depression severity in major depressive disorder. J Psychiatr Res. 2017;84:9–17. doi: 10.1016/j.jpsychires.2016.09.016 27673704

[16] Vinckier F, Gourion D, Mouchabac S. Anhedonia predicts poor psychosocial functioning: Results from a large cohort of patients treated for major depressive disorder by general practitioners. Eur Psychiatry. 2017;44:1–8. doi: 10.1016/j.eurpsy.2017.02.485 28535406

[17] Pelizza L, Ferrari A. Anhedonia in schizophrenia and major depression: state or trait?. Ann Gen Psychiatry. 2009;8:22. doi: 10.1186/1744-859X-8-22 19811665 PMC2764701

[18] Gillissie ES, Le GH, Rhee TG, Cao B, Rosenblat JD, Mansur RB, et al. Evaluating Anhedonia as a risk factor in suicidality: A meta-analysis. J Psychiatr Res. 2023;158:209–15. doi: 10.1016/j.jpsychires.2022.12.024 36603315

[19] Christensen MC, Ren H, Fagiolini A. Emotional blunting in patients with depression. Part IV: differences between patient and physician perceptions. Ann Gen Psychiatry. 2022;21(1):22. doi: 10.1186/s12991-022-00391-5 35733157 PMC9215037

[20] McMakin DL, Olino TM, Porta G, Dietz LJ, Emslie G, Clarke G, et al. Anhedonia predicts poorer recovery among youth with selective serotonin reuptake inhibitor treatment-resistant depression. J Am Acad Child Adolesc Psychiatry. 2012;51(4):404–11. doi: 10.1016/j.jaac.2012.01.011 22449646 PMC3536476

[21] Uher R, Payne JL, Pavlova B, Perlis RH. Major depressive disorder in DSM-5: implications for clinical practice and research of changes from DSM-IV. Depress Anxiety. 2014;31(6):459–71. doi: 10.1002/da.22217 24272961

[22] Vrieze E, Demyttenaere K, Bruffaerts R, Hermans D, Pizzagalli DA, Sienaert P, et al. Dimensions in major depressive disorder and their relevance for treatment outcome. J Affect Disord. 2014;155:35–41. doi: 10.1016/j.jad.2013.10.020 24210628 PMC3932031

[23] Serretti A. Anhedonia and Depressive Disorders. Clin Psychopharmacol Neurosci. 2023;21(3):401–9. doi: 10.9758/cpn.23.1086 37424409 PMC10335915

[24] Alsayednasser B, Widnall E, O’Mahen H, Wright K, Warren F, Ladwa A, et al. How well do Cognitive Behavioural Therapy and Behavioural Activation for depression repair anhedonia? A secondary analysis of the COBRA randomized controlled trial. Behav Res Ther. 2022;159:104185. doi: 10.1016/j.brat.2022.104185 36371903

[25] Baig-Ward KM, Jha MK, Trivedi MH. The Individual and Societal Burden of Treatment-Resistant Depression. Psychiatric Clinics of North America. 2023;46(2):211–26. doi: 10.1016/j.psc.2023.02.001 37149341 PMC11008705

[26] Perna G, Alciati A, Daccò S, Grassi M, Caldirola D. Personalized Psychiatry and Depression: The Role of Sociodemographic and Clinical Variables. Psychiatry Investig. 2020;17(3):193–206. doi: 10.30773/pi.2019.0289 32160691 PMC7113177

[27] Herr K, Berk M, Huang W-L, Kato T, Lee JG, Ng CG, et al. Anhedonia in Major Depressive Disorder: Prevalence and Treatment Expectations and Satisfaction with Treatment Goals Among Patients and Physicians in Asia-Pacific. Neuropsychiatr Dis Treat. 2024;20:2177–91. doi: 10.2147/NDT.S487747 39588177 PMC11586272

[28] Herr K, Berk M, Huang W-L, Kato T, Lee JG, Ng CG, et al. The Impact of Anhedonia on the Disease Burden of Major Depressive Disorder in the Asia-Pacific Region: A Cross-Sectional Real-World Study. Neuropsychopharmacol Rep. 2025;45(1):e70007. doi: 10.1002/npr2.70007 40011065 PMC11864854

[29] Kroenke K, Spitzer RL, Williams JB. The PHQ-9: validity of a brief depression severity measure. J Gen Intern Med. 2001;16(9):606–13. doi: 10.1046/j.1525-1497.2001.016009606.x 11556941 PMC1495268

[30] Snaith RP, Hamilton M, Morley S, Humayan A, Hargreaves D, Trigwell P. A scale for the assessment of hedonic tone the Snaith-Hamilton Pleasure Scale. Br J Psychiatry. 1995;167(1):99–103. doi: 10.1192/bjp.167.1.99 7551619

[31] Trøstheim M, Eikemo M, Meir R, Hansen I, Paul E, Kroll SL, et al. Assessment of Anhedonia in Adults With and Without Mental Illness: A Systematic Review and Meta-analysis. JAMA Netw Open. 2020;3(8):e2013233. doi: 10.1001/jamanetworkopen.2020.13233 32789515 PMC7116156

[32] United Nations D of E and SA Population Division. World Population Prospects 2022. New York: United Nations. 2022.

[33] Wang SM, Han CS, Lee SJ, Bahk WM, Pae CU. The Korean version of the Snaith-Hamilton pleasure scale: A preliminary study. Mood Emot. 2011;9:202–5.

[34] Nagayama H, Kubo S, Hatano T, Hamada S, Maeda T, Hasegawa T, et al. Validity and reliability assessment of a Japanese version of the Snaith-Hamilton pleasure scale. Intern Med. 2012;51(8):865–9. doi: 10.2169/internalmedicine.51.6718 22504240

[35] Leventhal AM, Unger JB, Audrain-McGovern J, Sussman S, Volk HE, Strong DR. Measuring Anhedonia in Adolescents: A Psychometric Analysis. J Pers Assess. 2015;97(5):506–14. doi: 10.1080/00223891.2015.1029072 25893676 PMC4545400

[36] Spitzer RL, Kroenke K, Williams JBW, Löwe B. A brief measure for assessing generalized anxiety disorder: the GAD-7. Arch Intern Med. 2006;166(10):1092–7. doi: 10.1001/archinte.166.10.1092 16717171

[37] McGahuey CA, Gelenberg AJ, Laukes CA, Moreno FA, Delgado PL, McKnight KM, et al. The Arizona Sexual Experience Scale (ASEX): reliability and validity. J Sex Marital Ther. 2000;26(1):25–40. doi: 10.1080/009262300278623 10693114

[38] Hays RD, Sherbourne CD, Mazel RM. The RAND 36-Item Health Survey 1.0. Health Econ. 1993;2(3):217–27. doi: 10.1002/hec.4730020305 8275167

[39] Samsa G, Edelman D, Rothman ML, Williams GR, Lipscomb J, Matchar D. Determining clinically important differences in health status measures: a general approach with illustration to the Health Utilities Index Mark II. Pharmacoeconomics. 1999;15(2):141–55. doi: 10.2165/00019053-199915020-00003 10351188

[40] Herdman M, Gudex C, Lloyd A, Janssen M, Kind P, Parkin D, et al. Development and preliminary testing of the new five-level version of EQ-5D (EQ-5D-5L). Qual Life Res. 2011;20(10):1727–36. doi: 10.1007/s11136-011-9903-x 21479777 PMC3220807

[41] Devlin N Prof. Dr., Parkin D, Janssen B. Methods for Analysing and Reporting EQ-5D Data. Cham (CH): Springer. 2020. doi: 10.1007/978-3-030-47622-9 33347096

[42] Reilly MC, Zbrozek AS, Dukes EM. The validity and reproducibility of a work productivity and activity impairment instrument. Pharmacoeconomics. 1993;4(5):353–65. doi: 10.2165/00019053-199304050-00006 10146874

[43] Franken IHA, Rassin E, Muris P. The assessment of anhedonia in clinical and non-clinical populations: further validation of the Snaith-Hamilton Pleasure Scale (SHAPS). J Affect Disord. 2007;99(1–3):83–9. doi: 10.1016/j.jad.2006.08.020 16996138

[44] Shankman SA, Katz AC, DeLizza AA, Sarapas C, Gorka SM, Campbell ML. The Different Facets of Anhedonia and Their Associations with Different Psychopathologies. Anhedonia: A Comprehensive Handbook Volume I. Springer Netherlands. 2014. p. 3–22. doi: 10.1007/978-94-017-8591-4_1

[45] Cheng C, Herr K, Jeon HJ, Kato T, Ng CH, Yang YK, et al. A Delphi consensus on clinical features, diagnosis and treatment of major depressive disorder patients with anhedonia amongst psychiatrists in the Asia-Pacific. Front Psychiatry. 2024;15:1338063. doi: 10.3389/fpsyt.2024.1338063 38463427 PMC10920342

[46] Cao B, Zhu J, Zuckerman H, Rosenblat JD, Brietzke E, Pan Z, et al. Pharmacological interventions targeting anhedonia in patients with major depressive disorder: A systematic review. Prog Neuropsychopharmacol Biol Psychiatry. 2019;92:109–17. doi: 10.1016/j.pnpbp.2019.01.002 30611836

[47] Gabbay V, Johnson AR, Alonso CM, Evans LK, Babb JS, Klein RG. Anhedonia, but not irritability, is associated with illness severity outcomes in adolescent major depression. J Child Adolesc Psychopharmacol. 2015;25(3):194–200. doi: 10.1089/cap.2014.0105 25802984 PMC4403015

[48] Chang C-M. What Do Patients Want in the Treatment of Major Depressive Disorder? Taiwan’s TAILOR Survey. Neurol Ther. 2023;12(Suppl 1):21–9. doi: 10.1007/s40120-023-00471-y 37115461 PMC10147885

[49] Kelly CA, Freeman KB, Schumacher JA. Treatment-resistant depression with anhedonia: Integrating clinical and preclinical approaches to investigate distinct phenotypes. Neurosci Biobehav Rev. 2022;136:104578. doi: 10.1016/j.neubiorev.2022.104578 35176319

[50] Seo JS, Bahk W-M, Woo YS, Park Y-M, Kim W, Jeong J-H, et al. Korean Medication Algorithm for Depressive Disorder 2021, Fourth Revision: An Executive Summary. Clin Psychopharmacol Neurosci. 2021;19(4):751–72. doi: 10.9758/cpn.2021.19.4.751 34690130 PMC8553538

[51] Whitton AE, Kumar P, Treadway MT, Rutherford AV, Ironside ML, Foti D, et al. Distinct profiles of anhedonia and reward processing and their prospective associations with quality of life among individuals with mood disorders. Mol Psychiatry. 2023;28(12):5272–81. doi: 10.1038/s41380-023-02165-1 37402852 PMC12835561

[52] Guajardo VD, Souza BP, Henriques SG, Lucia MC, Menezes PR, Martins MA, et al. Loss of interest, depressed mood and impact on the quality of life: cross-sectional survey. BMC Public Health. 2011;11:826. doi: 10.1186/1471-2458-11-826 22026632 PMC3213148

[53] Wong S, Le GH, Phan L, Rhee TG, Ho R, Meshkat S, et al. Effects of anhedonia on health-related quality of life and functional outcomes in major depressive disorder: A systematic review and meta-analysis. J Affect Disord. 2024;356:684–98. doi: 10.1016/j.jad.2024.04.086 38657767

[54] Thakurdesai A, Sawant N. A prospective study on sexual dysfunctions in depressed males and the response to treatment. Indian J Psychiatry. 2018;60(4):472–7. doi: 10.4103/psychiatry.IndianJPsychiatry_386_17 30581213 PMC6278224

[55] Rasmussen AL, Larsen SV, Ozenne B, Köhler-Forsberg K, Stenbæk DS, Jørgensen MB, et al. Sexual health and serotonin 4 receptor brain binding in unmedicated patients with depression-a NeuroPharm study. Transl Psychiatry. 2023;13(1):247. doi: 10.1038/s41398-023-02551-x 37414758 PMC10325956

[56] Vrieze E, Pizzagalli DA, Demyttenaere K, Hompes T, Sienaert P, de Boer P, et al. Reduced reward learning predicts outcome in major depressive disorder. Biol Psychiatry. 2013;73(7):639–45. doi: 10.1016/j.biopsych.2012.10.014 23228328 PMC3602158

[57] Borsini A, Wallis ASJ, Zunszain P, Pariante CM, Kempton MJ. Characterizing anhedonia: A systematic review of neuroimaging across the subtypes of reward processing deficits in depression. Cogn Affect Behav Neurosci. 2020;20(4):816–41. doi: 10.3758/s13415-020-00804-6 32472419 PMC7395022

[58] Menear M, Girard A, Dugas M, Gervais M, Gilbert M, Gagnon M-P. Personalized care planning and shared decision making in collaborative care programs for depression and anxiety disorders: A systematic review. PLoS One. 2022;17(6):e0268649. doi: 10.1371/journal.pone.0268649 35687610 PMC9187074

[59] Slade M. Implementing shared decision making in routine mental health care. World Psychiatry. 2017;16(2):146–53. doi: 10.1002/wps.20412 28498575 PMC5428178

[60] Choi H, Kim J-H, Park J-Y, Shim J-S, Lee J-G, Yoon H-Y, et al. Assessment of sexual dysfunction and determination of its risk factors in the Republic of Korea. Int J Gynaecol Obstet. 2014;125(1):60–4. doi: 10.1016/j.ijgo.2013.10.006 24462326

[61] Moreira ED Jr, Kim S-C, Glasser D, Gingell C. Sexual activity, prevalence of sexual problems, and associated help-seeking patterns in men and women aged 40-80 years in Korea: data from the Global Study of Sexual Attitudes and Behaviors (GSSAB). J Sex Med. 2006;3(2):201–11. doi: 10.1111/j.1743-6109.2006.00210.x 16490013

[62] Thakurta RG, Singh OP, Bhattacharya A, Mallick AK, Ray P, Sen S, et al. Nature of sexual dysfunctions in major depressive disorder and its impact on quality of life. Indian J Psychol Med. 2012;34(4):365–70. doi: 10.4103/0253-7176.108222 23723546 PMC3662135

[63] Winter J, Curtis K, Hu B, Clayton AH. Sexual dysfunction with major depressive disorder and antidepressant treatments: impact, assessment, and management. Expert Opin Drug Saf. 2022;21(7):913–30. doi: 10.1080/14740338.2022.2049753 35255754

