# Supplementary material for: Prevalence and burden of anhedonia among patients with major depressive disorder in South Korea: A cross-sectional, observational study
Source: PLoS One. 2025 Oct 27;20(10):e0334525. doi: 10.1371/journal.pone.0334525 (PMC12558494; doi:10.1371/journal.pone.0334525)
Supplement: S1 Table — (PDF) [file pone.0334525.s001.pdf]

S1 Table. Weighted correlation between Snaith-Hamilton pleasure scale (SHAPS) and 9-item patient health questionnaire (PHQ-9) scores

		PHQ-9 score	PHQ-9 item 1	PHQ-9 item 2	PHQ-9 item 3	PHQ-9 item 4	PHQ-9 item 5	PHQ-9 item 6	PHQ-9 item 7	PHQ-9 item 8	PHQ-9 item 9
**Total**											
SHAPS score	Pearson Correlation	0.42	0.30	0.31	0.25	0.24	0.20	0.28	0.22	0.19	0.29
	Sig. (2-tailed)	<0.001	<0.001	<0.001	<0.001	<0.001	<0.001	<0.001	<0.001	<0.001	<0.001
	r^2	0.18	0.09	0.10	0.06	0.06	0.04	0.08	0.05	0.04	0.08
**South Korea**											
SHAPS score	Pearson Correlation	0.08	0.07	0.01	0.03	0.04	0.05	-0.001	0.03	0.07	0.07
	Sig. (2-tailed)	0.093	0.173	0.811	0.506	0.386	0.294	0.983	0.478	0.139	0.127
	r^2	0.01	0.005	0.0001	0.001	0.002	0.003	0.000001	0.001	0.005	0.005

Note: Prevalence rates were weighted based on age and gender weighted using the UN population estimates for South Korea
PHQ-9, 9-item patient health questionnaire; SHAPS, Snaith-Hamilton pleasure scale; UN, United Nations.
