# Supplementary material for: Prevalence and burden of anhedonia among patients with major depressive disorder in South Korea: A cross-sectional, observational study
Source: PLoS One. 2025 Oct 27;20(10):e0334525. doi: 10.1371/journal.pone.0334525 (PMC12558494; doi:10.1371/journal.pone.0334525)
Supplement: S2 Table — (PDF) [file pone.0334525.s002.pdf]

S2 Table. Patient's characteristics (unweighted) among major depressive disorder (MDD) patients with anhedonia (MDD-ANH) and MDD patients without anhedonia (MDD non-ANH)

Variables	MDD-ANH (n=260)	MDD non-ANH (n=119)	p-value
**Sociodemographic characteristics**			
**Gender, n (%)**			
Male	151 (58.1)	76 (63.9)	0.2859
Female	109 (41.9)	43 (36.1)	
**Age, years, mean (SD)**	38.13 (10.73)	38.70 (9.58)	0.6222
**Age category, years, n (%)**			
18 to <25	23 (8.9)	5 (4.2)	0.4528
25 to <35	91 (35.0)	42 (35.3)	
35 to <45	83 (31.9)	41 (34.5)	
45 to <55	42 (16.2)	25 (21.0)	
55 to <65	18 (6.9)	5 (4.2)	
65 and older	3 (1.2)	1 (0.8)	
**Education, n (%)**			
Elementary school	1 (0.4)	0 (0.0)	0.33
Junior high school	2 (0.8)	1 (0.8)	
High school	46 (17.7)	14 (11.8)	
2-year college	38 (14.6)	12 (10.1)	
College	157 (60.4)	83 (69.8)	
Graduate school	16 (6.2)	8 (6.8)	
No school	0 (0.0)	1 (0.8)	
**Employment status, n (%)**			
Employed full time	170 (65.4)	87 (73.1)	0.1352
Self-employed	24 (9.2)	8 (6.7)	0.415
Employed part time	24 (9.2)	11 (9.2)	0.9968
Homemaker	11 (4.2)	5 (4.2)	0.9896
Retired	8 (3.1)	0 (0.0)	0.0609
Student	7 (2.7)	1 (0.8)	0.4439
Not employed, but looking for work	12 (4.6)	5 (4.2)	0.8567
Not employed and not looking for work	9 (3.5)	3 (2.5)	0.7601
**General health characteristics**			
**BMI, mean (SD)**	22.62 (4.19)	22.12 (3.60)	0.2599
**Frequency of smoking, n (%)**			
Everyday	139 (53.5)	65 (54.6)	0.0007
Some days	37 (14.2)	33 (27.7)	
Not at all	84 (32.3)	21 (17.7)	
**Frequency of consuming alcohol, n (%)**			
Every day	78 (30.0)	38 (31.9)	0.2791
Some days	145 (55.8)	71 (59.7)	
Not at all	37 (14.2)	10 (8.4)	

**Weekly exercise frequency, n (%)**			
More than 5 times a week	30 (11.5)	35 (29.4)	<0.0001
3-5 times a week	72 (27.7)	39 (32.8)	
1-2 times a week	76 (29.2)	29 (24.4)	
Very rarely or never	82 (31.5)	16 (13.5)	
**CCI, mean (SD)**	1.44 (2.66)	1.81 (3.31)	0.2877
**CCI Score Categories, n (%)**			
0	150 (57.7)	64 (53.8)	0.2041
1	37 (14.2)	11 (9.2)	
2	23 (8.9)	17 (14.3)	
3+	50 (19.2)	27 (22.7)	

Note: Prevalence rates were weighted based on age and gender weighted using the UN population estimates for South Korea.

ANH, anhedonia; BMI, body mass index; CCI, Charlson comorbidity index; MDD, major depressive disorder; MDD-ANH, MDD with anhedonia; MDD non-ANH, MDD without anhedonia; SD, standard deviation; UN, United Nation.
