# Supplementary material for: Prevalence and burden of anhedonia among patients with major depressive disorder in South Korea: A cross-sectional, observational study
Source: PLoS One. 2025 Oct 27;20(10):e0334525. doi: 10.1371/journal.pone.0334525 (PMC12558494; doi:10.1371/journal.pone.0334525)
Supplement: S3 Table — (PDF) [file pone.0334525.s003.pdf]

S3 Table. Physicians' clinical experience and perceived patient load with major depressive disorder (MDD)

Variables	n=60
Years practicing as a psychiatrist, years, mean (SD)	10.82 (5.57)
Percentage of time in direct patient care, %, mean (SD)	81.8 (16.4)
Number of MDD patients seen in the past month, mean (SD)	197.68 (182.04)
**Among physicians' patient caseload**	
**Perceived Severity of MDD among patients, %, mean (SD)**	
Mild	40.1 (17.6)
Moderate	39.0 (11.1)
Severe	20.9 (11.0)
Percentage of MDD patients perceived to have anhedonia, %, mean (SD)	37.5 (20.8)
**Perceived Severity of ANH among MDD-ANH patients, %, mean (SD)[a], n=43**	
Mild	29.6 (18.4)
Moderate	35.0 (13.2)
Severe	35.4 (22.7)

[a]0.3 (0.5)% (n=60) of patients did not know the breakdown of severity.
ANH, anhedonia; MDD, major depressive disorder; MDD-ANH, MDD with anhedonia; PHQ-9, 9-item patient health questionnaire; SD, standard deviation; SHAPS, Snaith-Hamilton pleasure scale.
