# Supplementary material for: Prevalence and burden of anhedonia among patients with major depressive disorder in South Korea: A cross-sectional, observational study
Source: PLoS One. 2025 Oct 27;20(10):e0334525. doi: 10.1371/journal.pone.0334525 (PMC12558494; doi:10.1371/journal.pone.0334525)
Supplement: S4 Table — (PDF) [file pone.0334525.s004.pdf]

S4 Table. Weighted healthcare resource utilization among major depressive disorder (MDD) patients with anhedonia (MDD-ANH) and MDD patients without anhedonia (MDD non-ANH)

**Variables**	**MDD-ANH (n=258), n (%)**	**MDD non-ANH (n=108), n (%)**	**p-value**
**Physician seen in the last 6 months**			
Emergency Medicine Specialist	64 (24.7)	44 (40.7)	0.0111
Psychiatrist	58 (22.3)	22 (20.7)	0.7499
Dentist	50 (19.3)	11 (10.2)	0.0654
Internist	49 (18.8)	25 (22.9)	0.4211
Psychologist / Therapist	45 (17.5)	9 (8.7)	0.0512
Pharmacist	39 (15.2)	6 (5.6)	0.0095
None of these	34 (13.1)	8 (7.7)	0.1909
Otolaryngologist (Ears, Nose, and Throat specialist)	30 (11.7)	4 (3.6)	0.0107
Dermatologist	30 (11.6)	10 (9.5)	0.6073
Ophthalmologist	29 (11.3)	5 (4.3)	0.0380
General Practitioner/Family Practitioner	29 (11.1)	12 (10.8)	0.9714
Endocrinologist	22 (8.5)	3 (2.8)	0.0560
Gastroenterologist	20 (7.9)	10 (9.1)	0.7267
Neurologist	20 (7.7)	4 (4.1)	0.1596
Orthopedist	20 (7.6)	3 (3.2)	0.1030
Cardiologist	16 (6.4)	1 (1.3)	0.0331
Allergist	15 (5.9)	7 (6.4)	0.8305
Korean medicine doctor	14 (5.4)	7 (6.1)	0.7951
Physical therapist	12 (4.5)	5 (4.4)	0.9723
Diabetologist	10 (3.8)	6 (6.0)	0.3228
Gynecologist	9 (3.5)	2 (1.7)	0.3710
Respiratory Therapist	8 (2.9)	4 (3.6)	0.8051
Pulmonologist	7 (2.8)	9 (8.1)	0.2209

Massage therapist	6 (2.5)	1 (0.6)	0.1645
Chiropractor	5 (2.1)	2 (2.3)	0.9252
Podiatrist	5 (1.9)	0 (0)	0.0003
Acupuncturist	5 (1.8)	10 (9.0)	0.0004
Hepatologist	4 (1.6)	0 (0)	0.0009
Nephrologist	4 (1.5)	2 (2.1)	0.6580
Urologist	4 (1.5)	1 (0.6)	0.4426
Plastic Surgeon	3 (1.1)	0 (0)	0.0060
Pharmacy assistant	3 (1.1)	2 (1.5)	0.6979
Oncologist	2 (0.7)	0 (0)	0.0390
Surgeon	1 (0.4)	2 (1.9)	0.1040
Optometrist	1 (0.3)	2 (2.1)	0.0333
Nutritionist	1 (0.3)	2 (1.9)	0.0468
Others	1 (0.3)	2 (1.9)	0.0468
Occupational therapist	1 (0.3)	1 (0.6)	0.5247
Infectious Disease Specialist/Infectologist (diseases such as HIV or Hepatitis)	0 (0)	3 (2.6)	<.0001
**Other healthcare services in the last 6 months**			
Pharmacy with health clinic (e.g., a walk-in clinic found in a retail store or pharmacy)	160 (61.9)	83 (76.7)	0.0162
Online pharmacy	42 (16.5)	21 (19.4)	0.5209
Telemedicine (e.g. use of text messages, e-mail, or software to provide clinical services virtually without an in-person visit)	29 (11.4)	7 (6.8)	0.1614
None of these	66 (25.5)	17 (15.5)	0.0629

Note: One participant visited each Geriatrician, Hematologist and Rheumatologist in MDD-ANH group while one participant in MDD non-ANH visited Homeopath.
ANH, anhedonia; MDD, major depressive disorder; MDD-ANH, MDD with anhedonia; MDD non-ANH, MDD without anhedonia.
